# Structural and Transport Properties of E-Beam Sintered Lanthanide Tungstates and Tungstates-Molybdates

**DOI:** 10.3390/nano12193282

**Published:** 2022-09-21

**Authors:** Vladislav Sadykov, Yuliya Bespalko, Ekaterina Sadovskaya, Tamara Krieger, Vladimir Belyaev, Nikita Eremeev, Mikhail Mikhailenko, Alexander Bryazgin, Mikhail Korobeynikov, Artem Ulihin, Nikolai Uvarov

**Affiliations:** 1 Federal Research Center, Boreskov Institute of Catalysis SB RAS, Akad. Laverntieva Ave. 5, 630090 Novosibirsk, Russia; 2 Institute of Solid State Chemistry and Mechanochemistry SB RAS, Kutateladze Str. 18, 630128 Novosibirsk, Russia; 3 Budker Institute of Nuclear Physics SB RAS, Akad. Laverntieva Ave. 11, 630090 Novosibirsk, Russia

**Keywords:** lanthanide tungstates, e-beam sintering, protonic conductivity, oxygen mobility

## Abstract

Lanthanide tungstates and molybdates are promising materials for hydrogen separation membranes due to their high protonic conductivity. A promising approach to fabricating ceramics based on these materials is radiation thermal sintering. The current work aims at studying the effect of radiation thermal sintering on the structural morphological and transport properties of (Nd,Ln)_5.5_(W,Mo)O_11.25–δ_ as promising materials for hydrogen separation membranes. The defect fluorite structure was shown to be preserved during radiation thermal sintering at 1100 °C. The presence of protons in hydrated samples was confirmed by TGA. According to four-electrode studies and the isotope exchange of oxygen with C^18^O_2_, the samples demonstrate a high proton conductivity and oxygen mobility. Residual porosity (up to 29%) observed for these samples can be dealt with during membrane preparation by adding sintering aids and/or metal alloys nanoparticles. Hence, sintering by e-beams can be applied to the manufacturing of hydrogen separation membranes based on these materials.

## 1. Introduction

Lanthanide tungstates and molybdates (Ln_28−x_M_x_O_54+δ_, Ln = La, Nd, Gd, Er, etc., M = Mo, W) as triple-conductive materials (protonic + oxide ionic + electronic conductivity) are promising for their application in hydrogen energy materials, including hydrogen separation membranes [1,2,3,4,5,6]. A high protonic conductivity is necessary for a hydrogen separation membrane to achieve high permeation fluxes [6,7,8]. A strong contribution of electronic conductivity is required for hydrogen transport across the membrane as well as avoiding its limitations by the coupled transport of electrons or holes [7,8]. In addition, oxide ionic conductivity is recommended to be high, since some proton transport mechanisms involve a jump between oxide anions and a movement of structural hydroxide anions [6,7]; moreover, additional hydrogen yield can be obtained by a water splitting reaction coupled with oxide anion transport across the membrane [8]. The high protonic conductivity of lanthanide tungstates and molybdates, along with electronic and, in some cases, oxide-ionic conductivity, allows high hydrogen permeation fluxes across the membranes based on such materials (up to ~10^−1^ mL H_2_ cm^−2^ min^−1^ at 1000 °C) [8,9,10,11,12,13,14].

To control the transport properties of such materials, the synthesis temperature can be varied in order to obtain highly conductive fluorite or bixbyite phases [3,4,5,6,15,16,17,18]. Along with this, the Ln site can be doped with various cations, such as other lanthanides [6,14,18] with the M site being able to include both tungsten and molybdenum as well as other cations [10,11,12] to further increase the protonic conductivity.

However, using lanthanide tungstates as materials for membranes or their functional (permselective) layers requires achieving a dense structure (with a relative density of at least 95% of a theoretical one) and forming defect fluorite or bixbyite polymorph with a high conductivity, which requires using sintering aids, high temperatures (1500–1600 °C) and extended timeframes (10–24 h) of thermal processing while using conventional sintering in a furnace (CS) for many compositions [3,5,8,10,11,19]. Hence, obtaining functional ceramics based on these materials is a separate problem. A promising approach to solve this problem is by using advanced sintering techniques, such as radiation thermal sintering using beams of electrons with a high intensity which allows us to carry out synthesis and sintering processes in reduced timeframes and at lower temperatures while obtaining products with a lesser porosity [6,20,21,22].

According to the authors’ previous studies [23,24], neodymium tungstates (Nd_5.5_WO_11.25−δ_) possess a high oxygen mobility (oxygen tracer diffusion coefficient up to ~10^−7^ at 700 °C) as well as a high protonic conductivity (~10^−5^–10^−4^ S/cm at 600 °C) with doping with La and Mo increasing the protonic conductivity (up to ~10^−4^–10^−3^ S/cm at 600 °C). This work aims at sintering materials based on neodymium tungstate by e-beams and characterization of their properties to elucidate whether this technique can be safely applied for the manufacturing of hydrogen separation membranes based on these materials. Lanthanide tungstates, including those doped with molybdenum (Nd_5.5_WO_11.25−δ_, (Nd_5/6_La_1/6_)_5.5_WO_11.25−δ_, and Nd_5.5_W_0.5_Mo_0.5_O_11.25−δ_) synthesized by mechanical activation, were sintered using e-beams for the first time. Structural, morphological features, proton content and transport characteristics of the samples obtained were studied using X-ray diffraction, the Archimedes’ technique, scanning electron microscopy, thermogravimetry analysis, four-electrode and isotope exchange of oxygen techniques.

## 2. Materials and Methods

Nd_5.5_WO_11.25−δ_ (NWO), (Nd_5/6_La_1/6_)_5.5_WO_11.25−δ_ (NLWO) and Nd_5.5_W_0.5_Mo_0.5_O_11.25−δ_ (NWMO) samples were synthesized using the mechanical activation technique [23]. Commercial powders of Nd_2_O_3_ (Novosibirsk rare earth metals plant, purity of 99.5%), La_2_O_3_ (Vekton, 99.999%) and MoO_3_ (Reachem, >99%) were used as starting materials. WO_3_ was obtained by calcination of tungsten acid (Vekton, >98%) at 300 °C. Mechanical activation was carried out using an AGO-2 planetary mill (CJSC NOVIC, Novosibirsk, Russia). Synthesis was carried out in steel shells with a volume of 150 mL at a rotation rate of 1200 rpm. ZrO_2_ balls with diameter of 1 cm were used as grinding objects with their mass ratio to material processed of 20:1. The process was carried out in the presence of propanol-2 for 20 min. According to elementary analysis, Fe admixture content did not exceed 0.02 wt.%.

To prepare samples for sintering, dried powders were pressed into pellets with a mass of 1.5 g, a diameter of 15 mm and a thickness of 1 mm at a pressure of 150 MPa.

Radiation thermal sintering (RTS) using e-beams was carried out using an ILU-6 accelerator [25]. Electrons’ impulses with 2.4 MeV energy, 328 mA pulse beam current, pulse duration ~600 μs, narrow scan, up to 25 Hz pulses frequency were used. The temperature of the samples was controlled using a Pt-Pt-Rh thermocouple (S-type) and FildPoint (National Instruments) controlling module. Processing temperature parameters were set using the controlling software of the accelerator. Power adjustment was carried out by changing the frequency of the pulses. The heating rate was 50 °C min^−1^, and after achieving the required temperature in the range of 1100–1300 °C, the samples were sintered for 30 min at a given temperature.

Phase composition and true density of the pelletized samples obtained were characterized by X-ray diffraction (XRD). XRD studies were carried out using a D8 Advance (Bruker, Ettlingen, Germany) diffractometer with a Cu Kα radiation source and LynxEye detector in 2*θ* range of 10–80° with 0.05° step.

Morphological characteristics of pelletized samples were studied by scanning electron microscopy (SEM) and Archimedes’ techniques. To prepare samples for SEM studies of the pellet surfaces, the samples were put into a cuvette then casted in the epoxide resin. The cuvettes were placed in a Struers filling machine, vacuumized and poured by preliminary prepared Struers EpoxiFix-20 epoxide resin. After resin hardening, the samples were ejected from the cuvettes with the excess of resin being removed using a Struers manual grinding machine. The samples were subsequently washed with water and ethanol and then dried. The particle size of the samples obtained was estimated by analyzing SEM images using Inkscape software. Additionally, the true density and porosity of the pelletized samples were studied by the Archimedes’ method.

For estimation of proton content and studying dehydration dynamics of RTS samples the thermogravimetry analysis (TGA) technique was used. The pelletized samples were preliminarily kept in distilled water at room temperature for 4 weeks, then dried in air at room temperature to remove water from the samples’ surface and residual pores. TGA studies were carried out using a STA 409 PC “LUXX” NETZSCH machine (Netzsch, Selb, Germany). The heating was carried out in N_2_ flow up to 900 °C with a ramp of 10 °C min^−1^; then the samples were exposed at 900 °C for 30 min.

The proton conductivity of the compact pelletized samples was determined in a humid air atmosphere by the 4-probe van der Pauw method in the galvanostatic mode with 4 ion-selective probes made of proton-conducting La_0.99_Ca_0.01_NbO_4_ ceramics. Ion-selective electrodes allow the determination of proton conductivity, since they completely block the electron current, while they are reversible electrodes for protons.

Oxygen mobility and surface reactivity were studied by the temperature-programmed isotope exchange (TPIE) of oxygen with C^18^O_2_ in a flow reactor. The samples (0.25–0.5 mm fraction) were loaded into quartz tube reactors (with an inner diameter of 3 mm). Pretreatment was carried out in He + 1% O_2_ flow (25 mL min^−1^) at 700 °C for 30 min. The isotope exchange was carried out in He + 1% C^18^O_2_ + 1% Ar flow (25 mL min^−1^) while heating from 50 to 800 °C with a constant ramp of 5 °C min^−1^. ^18^O atomic fraction (*α*) and C^16^O^18^O atomic fractions (*f*_16–18_) responses were analyzed in order to estimate isotope exchange kinetic parameters as described elsewhere [24].

## 3. Results and Discussion

### 3.1. Structure and Phase Composition

**Figure 1:**
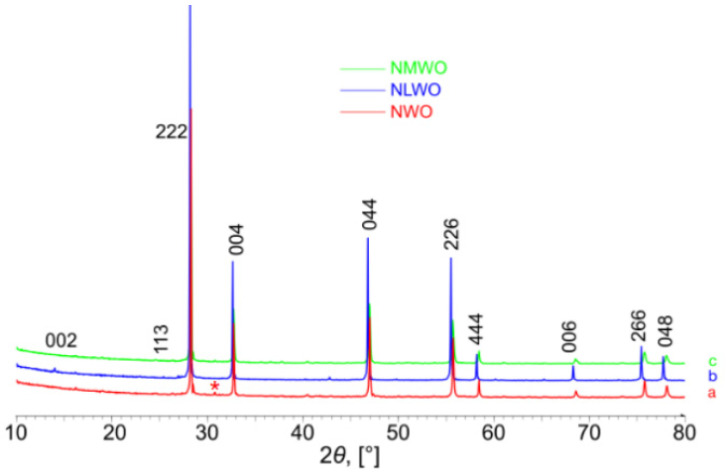
XRD patterns of samples synthesized by mechanical activation and sintered using electron beams at 1100 °C: (a) NWO, (b) NLWO, (c) NWMO. *—Nd_2_O_3_ [PDF 043-1023].

Figure 1 demonstrates XRD patterns of samples synthesized by the mechanical activation with various compositions (NWO, NLWO and NWMO) sintered at 1100 °C using RTS. All samples excluding NWO were single-phased. Samples have a defect fluorite structure with doubled unit cell (PDF 30-0687) [3,26]. According to XRD peak broadening, for all samples the grain sizes were >100 nm. The NWO sample contained Nd_2_O_3_ (PDF 043-1023) admixture. Obtaining single-phased NWO sample is probably possible while varying the Nd:W ratio [27,28,29,30], however, this is something to consider in future research. The unit cell parameter and volume of the samples studied as well as their true density values are given in Table 1. The unit cell parameter increases while substituting Nd cations with La ones, which is related to the larger radius of the La^3+^ cation [31]. Any significant variation of the unit cell parameter was not observed while doping the NWO sample with Mo cations.

Hence, from the point of view of phase composition, RTS conditions used in the current work did not lead to forming admixed phases and allowed us to obtain single-phase NLWO and NWMO samples. In the case of NWO, formation of an insignificant amount of Nd_2_O_3_ admixture was observed. This may be related to the accumulation of point defects while bombarding with the electron beam [20,32], which probably resulted in the local instability of the fluorite-like phase. The other reason is probably associated with the anisotropic effects while sintering due to the electrostatic repulsion of the electron beam and the charged particles of the sample [32,33,34]. Specific chemical processes during RTS, which are impossible to take place during conventional sintering in the furnace, can also affect the samples’ properties [33].

Cell parameters of the samples obtained by mechanical activation, RTS and conventional sintering (CS) are given in Table 1.

### 3.2. Morphological Characteristics

**Figure 2:**
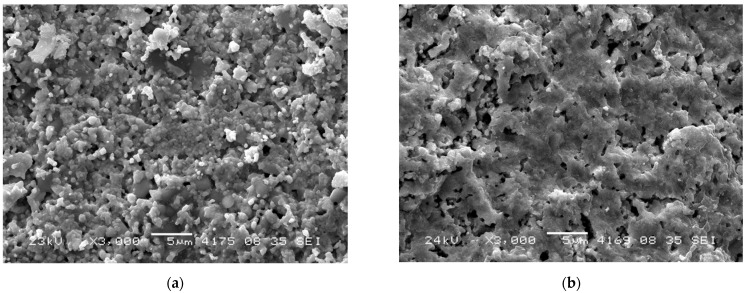
SEM micrographs of the NWO sample obtained by mechanical activation and RTS at 1100 °C acquired from the base (**a**) and the side (**b**) of the pellet.

**Figure 3:**
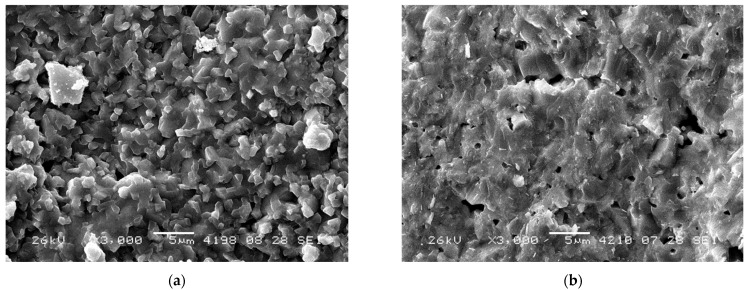
SEM micrographs of the NWMO sample obtained by mechanical activation and RTS at 1100 °C acquired from the base (**a**) and the side (**b**) of the pellet.

**Figure 4:**
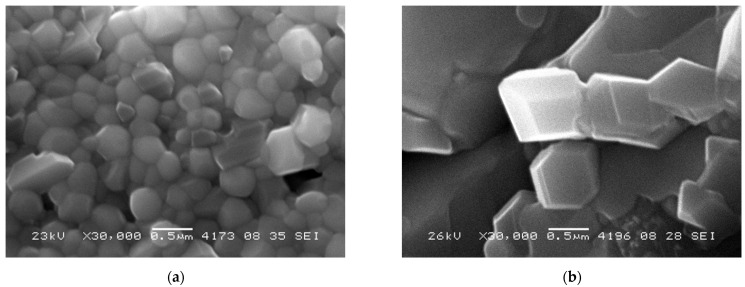
SEM micrographs of the NWO (**a**) and NWMO (**b**) samples obtained by mechanical activation and RTS at 1100 °C acquired from the base of the pellets.

Figure 2 and Figure 3 demonstrate low magnification SEM images of RTS samples prepared via mechanical activation. Images do not contain microcracks, which demonstrates the optimal sample sintering conditions. In this series, the samples have rather large particles with a size of ~100–500 nm (Figure 4), which form a network-like structure. This range agrees with the grain size estimated by XRD data analysis. Even larger particles (~700–900 nm) were obtained by conventional sintering at 1100 °C [23,24]. It is to be noted that the NWMO samples have larger particles compared to the NWO samples. A similar tendency was demonstrated in conventionally sintered samples [23,24].

Pores have an irregular shape with their characteristic size varying from a fraction of micrometer to a few micrometers. According to the SEM data, open pores are apparently present. The average porosity estimated according to Archimedes’ technique is equal to 21%, 29% and 21% for the NWO, NLWO and NWMO samples, respectively. Such rather high porosity can be explained by a poor sinterability due to a large particle size, a low RTS temperature as well as anisotropic effects while sintering which, in particular, can lead to the formation of additional porosity [34]. In comparison, the relative density of the NWO, NWMO and NLWO samples sintered conventionally at 1300 °C is 95%, 95% and 92%, respectively (Table 2) [23]. During membrane preparation, a higher residual porosity observed for these samples can be dealt with by adding sintering aids and/or metal alloy nanoparticles.

### 3.3. Proton Content and Conductivity

**Figure 5:**
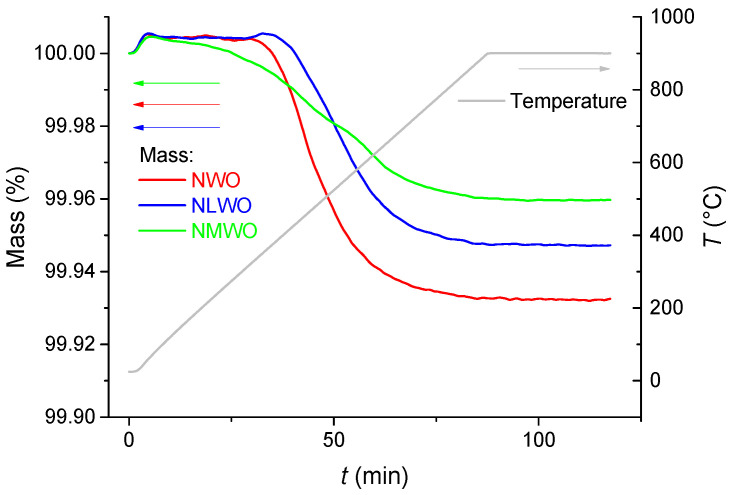
TGA curves of samples synthesized by mechanical activation and sintered using electron beams: NWO (red curve), NLWO (blue curve) and NWMO (green curve).

Figure 5 demonstrates TGA curves of the samples prepared by mechanical activation and RTS. A significant water removal during the TGA run was observed for all samples. The TGA curve of water removal from the NWMO sample had a shape of three steps (Figure 5 (green curve)), which is typical for proton conductors [6,35]. A low-temperature (30–300 °C) desorption is related to the removal of unbound water, residual water in the pores [35] as well as physically adsorbed water, including water located in the neighborhood of surface defects [6]. Removal of chemically adsorbed water started at temperatures above 300 °C. Chemisorbed water is apparently removed at 300–500 °C [6]. Along with this process, the structurally bonded water (hydroxyls) would start to be removed at these temperatures [35]. The third step is associated with the removal of structurally bound water and sample deprotonation. Full dehydration of the samples was achieved at 850–900 °C. For the NWO and NLWO samples, the weight loss was significantly higher, with TGA curves having a single step. This is probably related to a higher uniformity of proton mobility in the samples’ bulk.

Proton content in the samples of hydrated Ln tungstates can be estimated from the overall weight loss as a molar ratio of protons determined from the water amount desorbed during the TGA run and oxygen according to the sample stoichiometry (Table 3). Though doping should increase proton mobility [6,36], the opposite trends demonstrated by the proton content in Table 3 imply that water incorporation into proton conductors at room temperature is a complex process not determined completely by the proton mobility typical at intermediate temperatures.

Hence, proton content and water removal dynamics of samples of Ln tungstates synthesized by mechanical activation and processed by RTS were studied by the TGA technique. The samples were shown to possess properties typical of proton conductors. The better characteristics were demonstrated in the NWO sample.

**Figure 6:**
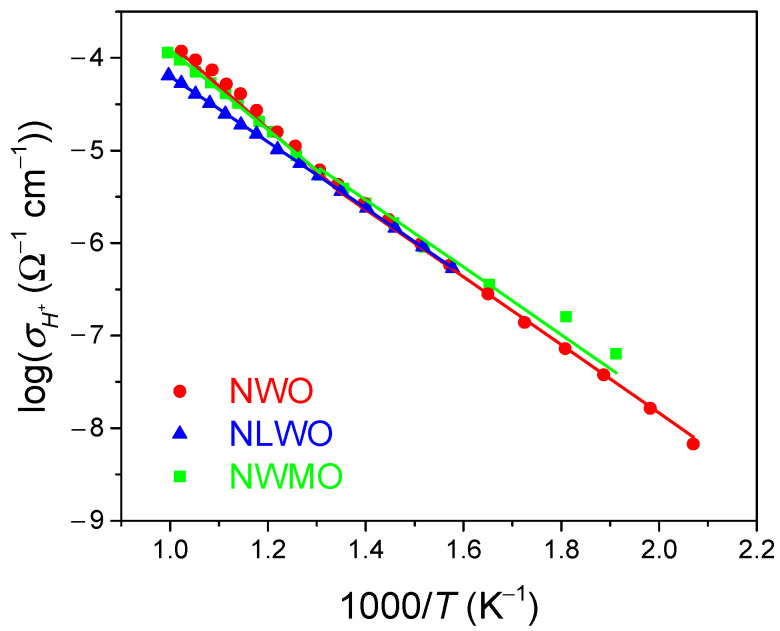
Protonic conductivity of samples synthesized by the mechanical activation and sintered using electron beams: NWO (red plot), NLWO (blue plot), NWMO (green plot).

According to van der Pauw method data (Figure 6), these samples demonstrated a high protonic conductivity (~10^−4^ Ω^−1^ cm^−1^ at 700 °C), which is close to the values acquired for CS samples sintered at 1350 °C [23]. Hence, RTS treatment allows the maintenance of protonic conductivity without its deterioration despite the remaining porosity. This can be explained by a higher disordering of the structure of e-beam sintered samples, which stabilizes their cubic fluorite structure, while CS samples sintered at 1350 °C have the tetragonal structure [23]. The effective activation energy for both the NWO and NWMO samples is ~75 kJ mol^−1^ at temperatures below 500 °C and ~95 kJ mol^−1^ at temperatures above 500 °C, while it was ~75 kJ mol^−1^ within the entire temperature range studied for the NLWO sample.

### 3.4. Oxygen Transport Characteristics

**Figure 7:**
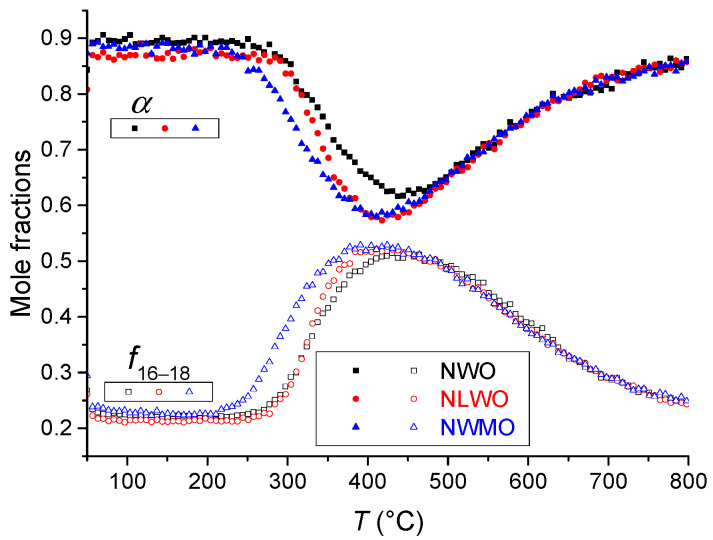
Temperature programmed isotope exchange of oxygen with C^18^O_2_ in the flow reactor for samples synthesized by mechanical activation and sintered by e-beams at 1100 °C: NWO (black squares), NLWO (red circles) and NWMO (blue triangles).

The oxygen mobility and surface reactivity of RTS samples were studied by TPIE C^18^O_2_ in the flow reactor. TPIE curves are given in Figure 7. The isotope label substitution during the TPIE run commenced at ~250 °C reaching extremes at ~400 °C. Almost all bulk oxygen (~96–100%) is involved in the isotope exchange process during the TPIE run, hence, demonstrating excellent characteristics in oxygen mobility and surface reactivity. Doping Nd_5.5_WO_11.25−δ_ with La or Mo slightly shifts TPIE peaks towards lower temperatures qualitatively demonstrating a higher oxygen mobility.

Similar to samples with the same compositions treated with CS at the same temperature [24], an interesting feature of the oxide ionic transport was revealed, namely, a fast oxygen diffusion along grain boundaries and a slower diffusion within the grains’ bulk (2D diffusion). Diffusion coefficient values along grain boundaries involving 5% of the overall oxygen are close for all samples (~10^−7^ cm^2^ s^−1^ at 500 °C, Table 4). A slower diffusion within the grains’ bulk (D^*^ in the range of 10^−13^–10^−15^ cm^2^ s^−1^ at 500 °C, Table 4) was revealed. As earlier demonstrated [24], for these complex oxides even in the bulk of grains three channels of oxygen diffusion can be distinguished. It is to be noted that the isotope exchange process is more intensive at temperatures below 400 °C for the NWMO sample compared to the NWO and NLWO ones. Similar to CS samples with the same compositions [24], it is apparently related to faster oxygen diffusion along grain boundaries for the NWMO sample compared with that of other samples (Table 4). This can be explained by the difference in the W–O and Mo–O binding energy as well as the effect of the Mo cation on the extended defect features, such as oxygen vacancy ordering or clustering [37,38], thin secondary phase film segregation along grain boundaries [39,40] and the probable effect on the porous structure [41]. At temperatures above 500 °C, the behavior of the isotope exchange process is almost the same for all three samples (Figure 7). This is probably due to the isotope substitution of oxygen contained in the grains’ bulk for which diffusivity is close for all three samples at these temperatures (Table 4).

## 4. Conclusions

In this work, the effect of radiation thermal sintering on the structural, morphological and transport properties of (Nd,Ln)_5.5_(W,Mo)O_11.25−δ_ as promising materials for hydrogen separation membranes was elucidated. The features of sintering (Nd,La)_5.5_(W,Mo)O_11.25−δ_ samples using e-beams were studied. There was no tetragonal splitting for radiation thermal sintered samples compared to samples sintered conventionally in the furnace, with peaks in XRD patterns of RTS samples being shifted towards lower angles (increased lattice parameters) compared with those of conventionally sintered ones. RTS samples synthesized by mechanical activation demonstrated certain water uptake according to TGA data which is typical for proton conductors. Their protonic conductivity is close to that of samples conventionally sintered at higher (1350 °C) temperatures despite a higher remaining porosity. According to isotope exchange data, a faster oxygen transport along grain boundaries and a slower oxygen transport within the grains’ bulk (2D diffusion) was demonstrated to be the feature of the oxides concerned. Hence, the sintering of these protonic conductors by e-beams does not deteriorate their transport properties, so it can be applied to the manufacturing of hydrogen separation membranes based on these materials, however, due to remaining residual porosity, using sintering aids is recommended.

## Figures and Tables

**Table 1:** The unit cell parameter (*a*), cell volume (*V*) and true density (*ρ*) of samples obtained by mechanical activation and sintered using electron beams (RTS) and conventional sintering (CS) [23].

Sample	Sintering Conditions	*a* (nm)	*V* (Å^3^)	*ρ* (g cm^−3^)	Reference
NWO	RTS, 1100 °C	1.0930	1305.39	5.89	This work
NWO	CS, 1100 °C	1.093	-	5.89	[23]
NWO	CS, 1300 °C	1.0915	-	7.1	[23]
NLWO	RTS, 1100 °C	1.0985	1318.93	5.80	This work
NLWO	CS, 1100 °C	1.098	-	5.80	[23]
NLWO	CS, 1300 °C	1.0943	-	6.8	[23]
NWMO	RTS, 1100 °C	1.0934	1307.18	5.66	This work
NWMO	CS, 1100 °C	1.098	-	5.66	[23]
NWMO	CS, 1300 °C	1.0906	-	7.0	[23]

**Table 2:** Morphological characteristics of the samples obtained by mechanical activation and sintered using electron beams (RTS) and conventional sintering (CS) [23].

Sample	Sintering Conditions	Particle Size (μm)	Relative Density (%)	Reference
NWO	RTS, 1100 °C	~0.3	79	This work
NWO	CS, 1100 °C	~0.7		[23]
NWO	CS, 1300 °C	~1	95	[23]
NLWO	RTS, 1100 °C		71	This work
NLWO	CS, 1100 °C	~0.8		[23]
NLWO	CS, 1300 °C	~1	92	[23]
NWMO	RTS, 1100 °C	~0.8	79	This work
NWMO	CS, 1100 °C	~0.9		[23]
NWMO	CS, 1300 °C	~1	95	[23]

**Table 3:** Molar ratio of the proton content to oxygen for the samples studied.

Sample	*n*(H^+^)/*n*(O^2−^)
NWO	0.00768
NLWO	0.00598
NWMO	0.00441

**Table 4:** Oxygen tracer diffusion coefficient values (D* (cm^2^ s^−1^)) at 500 °C for (Nd,La)_5.5_(W,Mo)O_11.25−δ_ samples sintered by RTS according to TPIE with C^18^O_2_ data.

Sample	Boundary Diffusion	Bulk Diffusion		
		Fast	Middle	Slow
NWO	4.1 × 10^−8^	2.1 × 10^−13^	4.5 × 10^−14^	1.1 × 10^−15^
NLWO	5.7 × 10^−8^	7.1 × 10^−14^	1.6 × 10^−14^	3.8 × 10^−16^
NWMO	5.9 × 10^−8^	2.9 × 10^−13^	5.2 × 10^−14^	9.0 × 10^−16^

## Data Availability

The data presented in this study are available on request from the corresponding author.

## Nomenclature

*a*	unit cell parameter (Å) or (nm);
*D**	oxygen tracer diffusion coefficient (cm^2^ s^−1^);
*f*_16–18_	C^16^O^18^O atomic fraction;
*n*(H^+^)/*n*(O^2−^)	molar ratio of the proton content to the oxygen;
*T*	temperature (°C) or (K);
*V*	unit cell volume (Å^3^);
**Greek letters**	
*α*	^18^O atomic fraction;
2*θ*	diffraction angle (°);
*ρ*	true density (g cm^−3^);
*σ_H^+^_*	protonic conductivity (Ω^−1^ cm^−1^).
